# Exploring the extent of source imaging: Recent advances in noninvasive electromagnetic brain imaging

**DOI:** 10.1016/j.cobme.2021.100277

**Published:** 2021-03-01

**Authors:** Abbas Sohrabpour, Bin He

**Affiliations:** Department of Biomedical Engineering, Carnegie Mellon University, United States

**Keywords:** Extent imaging, Electrophysiological source imaging, EEG, MEG, Sparsity, Bayesian framework

## Abstract

Electrophysiological source imaging (ESI) has been successfully employed in many brain imaging applications during the last 20 years. ESI estimates of underlying brain networks provide millisecond resolution of dynamic brain processes; yet, it remains to be a challenge to further improve the spatial resolution of ESI modality, in particular on its capability of imaging the extent of underlying brain sources. In this review, we discuss the recent developments in signal processing and machine learning that have made it possible to image the extent, i.e. size, of underlying brain sources noninvasively, using scalp electromagnetic measurements from electroencephalogram (EEG) and magnetoencephalogram (MEG) recordings.

## Introduction

Imaging brain electrical activity is of utmost importance for studying the normal and pathological brain processes [1]. An ideal imaging modality would offer a high spatial and temporal resolution, as well as a whole-brain coverage. Additionally, for such a brain imaging modality to be available for wide use in studying human brains, this modality should be preferably noninvasive [2]. Major candidates among imaging modalities include EEG and MEG source imaging, functional magnetic resonance imaging (fMRI), positron emission tomography (PET), single-photon emission computed tomography (SPECT), and functional near-infrared spectroscopy (fNIRS). Among these modalities, it is only EEG and MEG that provide a truly noninvasive and direct measurement of the underlying brain electrical activity in a high temporal resolution, as opposed to secondary effects from the neuronal hemodynamic response [3]. The spatial resolution of EEG and MEG are, however, limited in their raw and unprocessed form, i.e. as measured on the scalp. Electrophysiological source imaging (ESI) techniques, on the other hand, resolve this issue to a major extent and attempt to remove the volume conduction effect and mixing of sources at sensor-level measurements by estimating the distribution of dynamic brain sources using mathematical and biophysical principles [4].

It is, however, of great importance and relevance to investigate these questions regarding the efficacy of the ESI framework: how good are ESI estimates? Are such estimates capable of providing accurate information about source location and size? Can they estimate the temporal dynamics of brain activities? Can they be used effectively to address real clinical or research needs? The answer to these questions, as we will attempt to address in the following, is positive, as the ever-changing frontiers of research expand our knowledge at an unprecedented pace.

## Basics of electrophysiological source imaging

The process of estimating underlying brain electrical activity from scalp electromagnetic measurements, such as EEG and MEG, is referred to as ESI. Basically, brain electrical activities are modeled as current density dipoles, and the distribution of these dipoles in the brain is estimated from the electromagnetic fields they generate at EEG/MEG sensors [1,2]. ESI is basically an inverse problem that is inherently ill-posed as the number of unknowns is far greater than the number of measurements. However, ESI algorithms presume *a priori* properties and structure about the underlying sources and the brain-head tissue, i.e. spatial, spectral, and/or temporal features, to efficiently solve this problem. Some of the well-established ESI algorithms that were based on minimizing the L_2_-norm, such as the minimum norm estimate [5], sLORETA [6], and beamforming methods [7,8], have been proven efficient in solving this problem (please refer to Ref. [1] for a recent and comprehensive review on ESI methods and algorithms).

In brief, ESI algorithms provide estimates of underlying sources that satisfy two main conditions; first, they fit recorded measurements (at the sensor), which is represented with a fitting term in the corresponding optimization problem, and second, they satisfy *a priori* constraints such as having minimal norm or being sparse (at source), which is captured in the regularization term in the corresponding optimization formulation of the problem. The regularization terms are fundamental in forming solutions that satisfy certain properties at the source level, for instance, if they can model extended sources or are spatially smooth like most L_2_-norm estimates. Careful modeling of *a priori* knowledge via the regularization term, as we will discuss, is what gives more recently developed ESI methods a strong capability in estimating underlying brain sources with higher spatial resolution compared to more conventional approaches.

## Challenges that ESI research faces

One of the major challenges that presents itself to ESI research is the moderate spatial resolution of the estimated ESI solutions. Due to the ESI problem being an under-determined optimization problem, i.e. number of unknowns being larger than measurements, achieving a higher spatial resolution is challenging. Specifically, conventional algorithms that rely heavily on L_2_-norm estimates achieve robust inverse imaging at the expense of imposing overly smooth distributions in the source space. Two notable exceptions are the robust beamforming algorithm [9] and the ExSo-MUSIC algorithm [10]. These algorithms, while showing more sensitivity to the extent of underlying sources, still require subjective thresholding to separate signal from background. Estimating the extent and size of underlying sources from noninvasive measurements posed a major challenge for ESI research for many years. Extent estimation is quite important for a multitude of applications, including brain mapping, i.e. mapping the somatosensory cortex and identifying pathological networks such as the identification of the epileptogenic zone in focal epilepsy patients, to name a few examples.

Another major challenge that ESI algorithms face is the matter of estimating sources that are deeply located in the brain tissue. Signal strength quickly falls off with distance; thus, deeper sources typically produce weaker signals at the scalp [1]. This makes the estimation of deeper sources more challenging, reducing the accuracy and precision of localization for such sources.

In this review, we focus on the former challenge that is the spatial resolution of ESI estimates, and more specifically, estimating the extent of underlying sources from scalp measurements. We will, however, briefly mention how modern signal processing and computational tools have come to the rescue and alleviated the deep source issue, even when their major goal is to find a solution to the source extent estimation problem and not necessarily alleviating the imprecision of deep source estimates.

## Extent estimation with ESI – is it possible?

The key question in extent imaging is the following: How can information such as the extent of underlying brain sources be extracted from seemingly limited scalp measurements? In other words, how can an under-determined problem be exploited to derive such detailed and accurate information? The simple answer is that underlying brain sources have structures (or we think such structures exist), that once modeled in the form of *a priori* knowledge in the regularization term, can extract detailed information about the structure of underlying sources. In other words, if the redundancies of underlying sources are modeled, the limited measurements might be more than enough to estimate the extent of underlying sources. Put differently, the number of unknowns is not as high as we might have initially intuited.

It is common knowledge that sparse signals, i.e. vectors or matrices that have many zero elements and a small number/ratio of nonzero elements, can be recovered with the limited number of measurements proportional to this sparsity factor, commonly referred to as compressive sensing [11]. This means that if the underlying brain activity can be represented as a sparse vector, it can be recovered easily. Some of the earlier works in incorporating sparse signal recovery algorithms such as FOCUSS [12] and MCE [13] were developed based on this idea; however, many studies have suggested that underlying brain sources with detectable EEG signals at the scalp are not sparse and actually involve an extended cortical area [14,15]. On the other hand, the initial success of these algorithms (we will refer to such methods as sparse methods in the remainder of this paper) in localizing brain sources, e.g. source distributions’ centers of mass, is encouraging; indicating that precise information about underlying sources can be recovered if the ESI algorithm properly models such structures. How can such methods be implemented to model EEG sources’ redundancies while we know these sources are not sparse?

## Directly modeling sparsity – sparse methods

**Figure 1:**
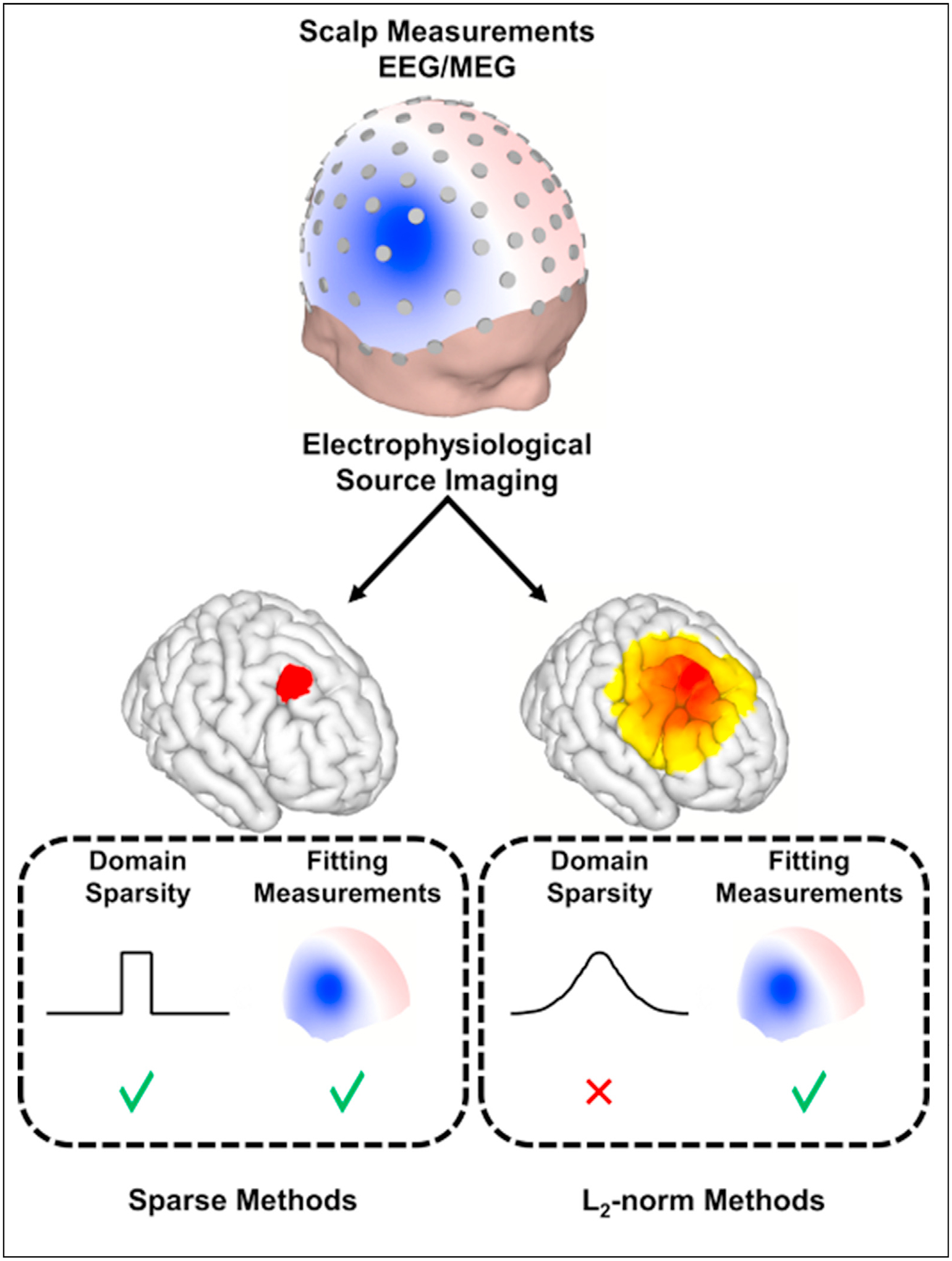
Retrieving Source Extent with Sparsity Assumptions. ESI approaches that model source redundancies, i.e. sparsity in the appropriate domain, can obtain extended sources while fitting the measurements. Conventional ESI algorithms rely heavily on L2-norm methods that produce heavily dispersed and overly smooth solutions. In the sparse example provided here, while the source itself is not sparse, its edges are sparse. In other words, the source is focally extended. Methods such as IRES can estimate such sources by modeling the edge sparsity, as well as applying an iterative reweighting strategy to estimate source extents objectively.

While underlying brain sources might not exactly be sparse, these sources might be represented sparsely once transformed into another domain; for instance, if a minimum of cortical tissue is necessary to be activated for its activity to be detected at EEG/MEG sensors, we might assume that it is a piecewise homogeneous distribution [16]. This means that while the source itself is not sparse, the gradient of the source, i.e. its edges, is sparse. The sparsity can be enforced in multiple domains, as well as a single domain, enforcing the gradient and the solution to be sparse, simultaneously [17]. This concept is schematically depicted in Figure 1.

Early works in developing sparse methods enforced sparsity on solution’s gradient [18], Laplacian [19], or even a combination of multiple domains [20] to acquire extended sources. These early works demonstrated the promise of sparse methods in estimating extended sources. However, the source extent could not be objectively determined as a background activity, which looked like noise, was present, and typically, was discarded with subjective thresholds. Later, Babadi et al. [21] proposed a greedy pursuit algorithm to solve this problem. They parceled the cortex into nonoverlapping regions and pursued to select the region that best fits the scalp measurements, removed the effect of this source, and repeated the process while imposing a sparse solution, i.e. only selecting a limited number of regions. Additionally, after an initial solution was obtained, each region was further divided into smaller regions, and the process was repeated for the smaller regions to refine the solution, similar to a multiresolution approach [22]. This work was later followed up by Krishnaswamy et al. to provide a systematic framework to find extended solutions and estimate the source extent [23]; however, this approach has a major limitation that is the *a priori* knowledge of sparsity level, or basically when to stop pursuing the best fit for the data, for avoiding overfitting and still obtaining a sparse solution. The manner in which parcellation is performed might also affect solutions, as it is not unique.

**Figure 2:**
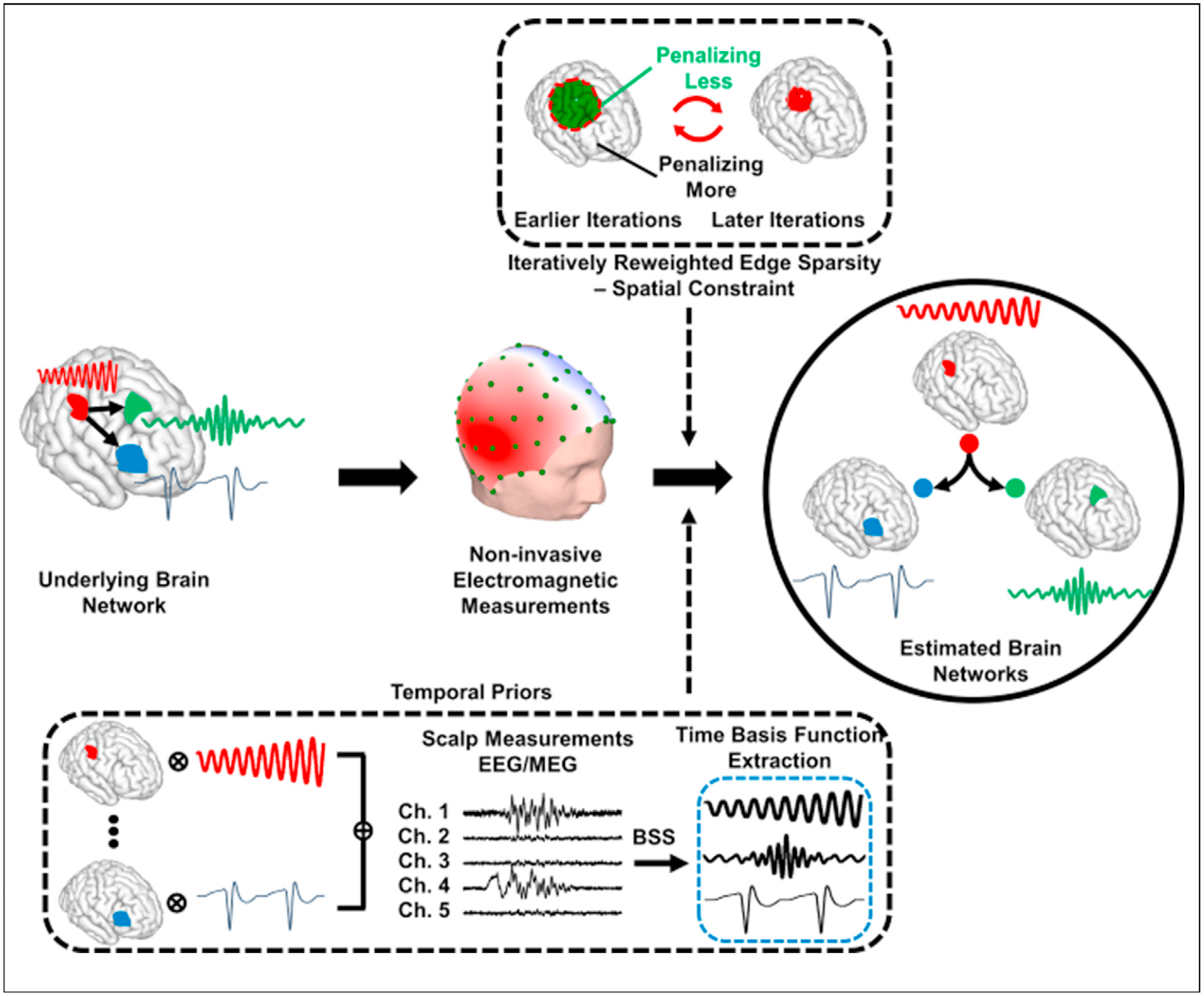
Modeling Brain Networks as Spatiotemporal Processes. Brain networks are modeled as focally extended sources that vary over time. The net-effect of these dynamics are recorded in EEG/MEG. Blind source separation (BSS) techniques applied to these measurements can delineate these underlying dynamics and serve as a temporal prior in the imaging algorithm. Spatial constraints that enforce the edge sparsity, i.e. clear distinction of activity and background noise, can be ensured by applying an iterative reweighting scheme in a data-driven manner to guarantee focally extended sources. Combining these data-driven priors into the imaging module, we can estimate underlying brain networks, the nodes, and internodal connectivity (links) of these networks. Reproduced with permission from Ref. [25].

Sohrabpour et al. [16] proposed IRES that is a fully data-driven method for estimating the extent of underlying sources. This method enforces sparsity on the solution, as well as its gradient, to ensure a focally extended source. Some literature suggests that this might be a good model for underlying brain sources [15]. Additionally, an iterative reweighting schedule is adopted that penalizes solutions that contain sources with small amplitudes [24]. Iteratively running the algorithms, the solution automatically sets background activity to zero and converges, in only a few iterations, to focally extended sources without the need for any posthoc thresholding algorithms, subjective or data-driven. IRES was later developed to handle spatiotemporal processes such as ictal activities captured in scalp EEG recordings, named FAST-IRES [25]. The data-driven approach proposed in IRES is capable of estimating the extent of underlying sources with high precision without the need for subjective thresholds. The objective and data-driven approach adopted in IRES for extent estimation, to our knowledge, is quite unique in the literature and has inspired some Bayesian approaches to even adopt its strategy [26,27]. Figure 2 schematically depicts the concepts on which FAST-IRES is based. The idea of spatial and temporal priors or (modeling *a priori* knowledge) is quite general and applies to most algorithms.

## The Bayesian framework

The Bayesian framework is a general approach in signal processing, machine learning, and optimization that can be used to solve many optimization problems. The underlying processes, brain sources in ESI problem, are modeled as stochastic phenomena with assumed probability distributions, referred to as priors. These priors are related to the measurements through a process that is determined by the physical laws governing the system under study, i.e. through the lead field and volume conduction in the ESI problem. The posterior distributions defined as the conditional probability of brain sources given scalp measurements are then updated repeatedly based on the priors and measurements, according to the Bayes rule, until the process converges to a solution [28]. Many conventional ESI algorithms such as minimum norm [5], sLORERTA [6], and FOCUSS [12] can be recast within the Bayesian framework [29]. The Bayesian framework is a powerful framework where all prior assumptions can be explicitly modeled through probability distributions and hyper-parameters. One of the potential issues of Bayesian approaches is the number of hyper-parameters that need to be fitted based on the data. This translates in the ESI problem to the many source covariances (for each source location) that need to be tuned to fit the measurements. Bayesian methods have also benefitted from sparse priors that induce or encourage sparse solutions or hyper-parameters [30], fitting hyperparameters efficiently. Careful choosing of such priors can result in the automatic relevance determination (ARD) of sources or hyperparameters of interest [31]. An automatic pruning process that can potentially discard irrelevant hyper-parameters and only keep the minimum number of hyperparameters needed to fit the data. As we will discuss in the following, these methods are powerful but need more careful selection and pruning to actually be able to estimate the extent of underlying sources.

Among the earlier ESI approaches within the Bayesian framework, the maximum entropy on the mean (MEM) has been quite successful [32]. The MEM approach which later, developed into coherent MEM (cMEM) [33,34], essentially tries to find the solution that best fits the data while having the least Kullback-Leibler (KL) divergence (also known as v-entropy) to a reference distribution, in effect forcing the obtained solution to have a similar distribution to that of the reference distribution, while fitting the measurements. The manner in which the reference distribution is defined in MEM assumes that different regions in the brain (needs to preparcellate the brain) are active with a probability (that will be estimated from data), and if they are activated, their activities assume a Gaussian distribution. The cMEM approach is capable of estimating extended because of the manner in which its reference distribution is defined; however, subjective or posthoc thresholding techniques such as Otsu’s method are required to estimate the source extent [34]. The MEM-family algorithms have been shown to be useful in estimating epilepsy sources from EEG and MEG measurements [35–37].

Another issue that Bayesian techniques face is that due to the alternating nature of updating their variables and hyperparameters in their optimization algorithms, the shape of the underlying sources’ covariances needs to be determined *a priori* so that the ARD process can later prune out irrelevant candidates and select relevant covariance matrices that best fit the data. This means that in order to estimate possible source covariances, these methods employ parcellation techniques to separate the brain into nonoverlapping regions to calculate the covariance among possible source patterns, typically in a hierarchical manner [34,38]. The sensor noise covariance needs to be known or estimated from baseline activities. Recently, a new algorithm from the Champaign family has incorporated this step into its optimization process [39]. However, Bayesian methods rely on accurate modeling of source covariances to function well and typically rely on parcellating the brain in an *a priori* manner or have to more explicitly model variables such as solution’s gradient into their model to successfully obtain extended solutions [26].

Interestingly, using a hierarchical Bayesian approach in which the brain is preparcellated into nonoverlapping regions [38] and subsequently penalizing sources based on cluster distances [40], it is possible to achieve spatially coherent extended solutions that approximate the extent of underlying sources. Thresholds still need to be applied to these solutions to estimate the extent.

Inspired by the idea proposed in IRES, some recent works in the Bayesian realm have adopted the approach of directly modeling the gradient of solutions, i.e. solution edges, with sparse priors [26]. One work went as far as applying an iterative reweighting scheme, similar to IRES, to clear background activity and estimate the extent [27]. To our knowledge, this is the only Bayesian ESI algorithm that does not need subjective or posthoc thresholds to estimate source extent, albeit following ideas similar to the IRES idea. In addition to modeling extended sources with spatial priors, Bayesian methods that produce spatially extended solutions can model the underlying sources’ temporal dynamics, as well. Notably, the sources’ temporal activities have been modeled with Markov random fields [41–43]. Moreover, modeling spatiotemporal priors adopting a matrix factorization approach [44] or Graph Theory concepts [45] has been attempted as well. These approaches ensured that underlying brain activities did not change drastically from time to time or suddenly disappear at a spatial location and emerge at another point on the cortex.

The Bayesian framework has demonstrated the importance of carefully modeling spatiotemporal *a priori* knowledge into the optimization framework to improve the spatial resolution of estimated solutions. Bayesian methods seem to be highly inspired by sparse methods and concepts in forming and solving the ESI problem. However, it seems that the more successful Bayesian methods discussed here have adopted similar strategies to IRES and FAST-IRES, such as iterative reweighting and directly modeling source gradients with sparse priors.

## Applications of extent imaging

Estimating the extent of underlying brain sources is important for brain mapping and imaging studies, as well as determining pathological networks such as epilepsy [46]. We will briefly discuss some of the applications of these recent algorithms, specifically focusing on somatosensory cortical mapping [39,40,44], auditory processing and language comprehension [45,47], and epilepsy imaging [25,48].

**Figure 3:**
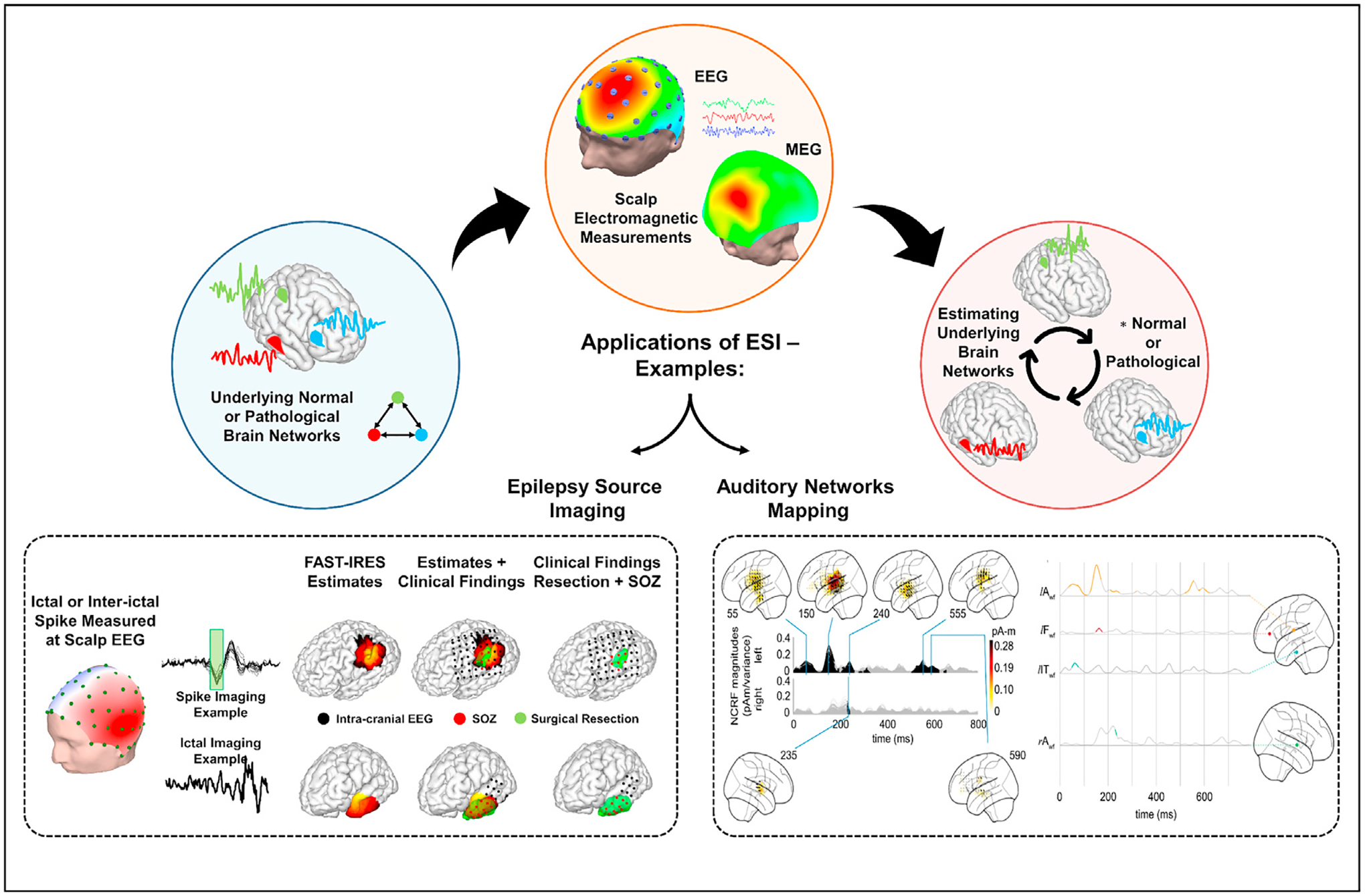
Examples of Extent Imaging and Its Application in Epilepsy Network Imaging (Pathological) and Auditory Networks Imaging (Normal). Electrophysiological source imaging techniques can be used to estimate the location, extent, and temporal dynamics of underlying brain networks, whether those networks pertain to normal networks or pathological processes. (lower row – left panel) An example of applying FAST-IRES to ictal and interictal signals recorded at scalp EEG of focal epilepsy patients is presented. Adapted from Ref. [25]. (lower row – right panel) An example of imaging auditory networks from MEG recording in healthy human subjects is also presented. This particular example demonstrates the results of analyzing semantic structures of auditory signals presented to subjects. From Ref. [47], with permission.

Imaging interictal discharges and ictal activities is important in determining the epileptogenic tissue in focal epilepsy patients [46]. Determining the location and extent of these abnormal tissues can be important in planning for treatment and possibly surgical resection of these tissues in focal epilepsy patients suffering from medically intractable seizures. In a recent study in 36 epilepsy patients, we showed how successful FAST-IRES is in determining the epileptogenic tissue from ictal and interictal signals [25]. Other studies using Bayesian methods have been demonstrated to be useful in determining the underlying epilepsy networks, as well [48,49]. Figure 3 shows how FAST-IRES has been applied to epilepsy network imaging.

Extent imaging can be employed to study healthy brain networks, such as noninvasively mapping the somatosensory cortex [39,40,44] or studying auditory processing and language comprehension in awake subjects [47]. In a recent study from Das et al. [47], in a continuous auditory stimulus paradigm, the auditory circuits and networks were studied from MEG measurements. The estimates from this Bayesian method were able to show lower order auditory networks and higher order semantic and language processing areas in a spatiotemporal manner where the extent of different auditory processing circuits could be determined from MEG measurements in addition to their temporal dynamics. Figure 3 provides an example from this work. Extent estimation can be used in many applications to determine the extent of underlying brain processes, as well as their location and temporal dynamics, an important feature for functional imaging of brain networks.

Note that many source imaging software and techniques are readily available to the public for free or via commercial entities. Some of the more famous open-source software and toolboxes include Brainstorm [50], eConnectome [51], Fieldtrip [52], MNE [53], and NUTMEG [54], to name a few. Commercial software packages such as CURRY (Compumedics, Charlotte, NC) and BESA (Brain Vision, Morrisville, NC) provide source imaging algorithms, as well. These platforms allow for ESI techniques and algorithms to be employed widely in basic and clinical neuroscience research. An important direction for future research is to further improve the robustness of ESI technology so that it can be broadly used in clinical settings with minimum training and intervention by the operators of these systems.

## The deep source problem

Imaging deep sources is one of the major challenges for most ESI approaches. It has been reported recently that deep brain sources can be reliably detected from EEG and MEG measurements [55,56]. A recent work developing sparse ESI methods showed how subcortical activities can be estimated even when stronger cortical activities are present [23]. This work suggests that if sources satisfy some sparsity conditions, they can be retrieved efficiently. Intuitively this can be explained by noting that deeper sources produce different EEG/MEG signals at the sensor level compared to cortical sources. For instance, the distance (to sensors) and geometry of deeper sources are quite different from cortical sources, and hence, the signals recorded from these sources at sensors are different from cortical sources. If cortical sources are dense, superficial sources may explain most of the signals recorded at sensors. On the other hand, sparse sources with limited activation extents produce distinct signals at sensors, allowing deep and superficial sources to be separated more successfully. Our own recent study contends with these findings and suggests that deep activity rising from parahippocampal gyrus can be estimated from scalp EEG using sparsity-based imaging algorithms [25]. These findings and observations suggest that the sparse framework might be able to tackle this challenge, as well as extent estimation, and warrants further investigation in the future.

## Conclusion and outlook

In this short review, we have strived to demonstrate the value and utility of ESI algorithms in brain source imaging research and clinical applications, specifically in terms of estimating the extent of underlying brain sources. While ESI is an under-determined problem, this does not imply that precise and high spatial resolution estimates cannot be derived from noninvasive scalp measurements. On the contrary, we showed how the present algorithms in the literature, either by explicitly using sparse models or adopting a Bayesian approach, have been capable of extracting detailed information about the extent of underlying sources, hugely improving the spatial resolution of underlying sources (refer to Table 1 for a summary). This is only possible as appropriate prior knowledge about underlying sources’ properties and spatiotemporal structures were considered in modeling the ESI problem.

Multimodal neuroimaging such as simultaneous EEG-fMRI [57–59], EEG-fNIRS [60], or MEG-fNIRS [61] could provide useful information about the underlying sources’ properties that could potentially inform the priors of source imaging algorithms. Multimodal neuroimaging has the merit of combining the strengths of individual modalities, e.g., a high spatial resolution of fMRI and high temporal resolution of EEG/MEG, to obtain complementary information about the underlying brain activity, e.g., spatial distribution or temporal dynamics [1]. However, resolving potential inconsistent sources in different modalities, which may be observable in one modality and hidden in another, remains to be a challenge for future research in multimodal neuroimaging.

We would like to bring the reader’s attention to the fact that what noninvasive techniques might lack in the number of measurements, must (and can) be efficiently substituted for by intelligent computational models and prior knowledge derived from biophysical principles. We believe that the future bears much more interesting research in the field of Electrophysiological Source Imaging.

## Figures and Tables

**Table 1:** A summary of recent ESI algorithms capable of extent estimation.

Family	Algorithm category and related paper
**Sparse Methods**	**Greedy Pursuit** – Babadi et al. (2014), Krishnaswamy et al. (2017) {[21,23]} **Domain Sparsity with iterative reweighting** – Sohrabpour et al. (2016, 2020) {[16,25]}
**Bayesian Methods**	**MEM** – Grova et al. (2006), Chowdhury et al. (2013, 2016) {[32–34]} **Markov Random Field Modeling** – Liu et al. (2016), Hansen & Hansen (2016), Pirondini et al. (2018) {[41–43]} **Hierarchical** – Cai et al. (2018, 2019, 2021) {[38–40]} **Variational Sparsity** – Liu et al. (2018, 2020) {[26,27]} **Spatiotemporal Matrix Factorization** – Liu et al. (2019) – {[44]} **Probabilistic Structure Learning** – Liu et al. (2020) {[45]}
